# A Case of Relapse Pseudomonas aeruginosa Tricuspid Valve Endocarditis After AngioVac Vegectomy and Antibiotic Treatment in a Patient Using Intravenous Drugs

**DOI:** 10.7759/cureus.65867

**Published:** 2024-07-31

**Authors:** Parveen Gaba, Chidi D Okoroafor, Madhu Suryadevara, Maikel Tawadrous

**Affiliations:** 1 Infectious Disease, Newark Beth Israel Medical Center, Newark, USA; 2 Internal Medicine, Trinity Health of New England, New Haven, USA; 3 Medicine, St. George's University, True Blue, GRD

**Keywords:** angiovac and infective endocarditis, intravenous drug use (ivdu), relapse of pseudomonas bacteremia, pseudomonas aeruginosa, infective endocarditis

## Abstract

Infective endocarditis (IE) is a bloodstream infection affecting the valves of the heart. IE is highly associated with morbidity and mortality if not properly managed. *Pseudomonas aeruginosa *(*P. aeruginosa*) as a cause of IE is extremely rare. This is a case of IE involving a male patient with a history of intravenous drug use (IVDU), secondary to *P. aeruginosa, *with associated relapse of bacteremia and native tricuspid valve endocarditis, complicated by septic pulmonary emboli, despite undergoing recent vegetation debulking using the AngioVac system (AngioDynamics, Inc., New York, USA) along with six weeks of IV antibiotics and no IVDU since then being on treatment.

## Introduction

Infective endocarditis (IE) has an annual incidence rate of 3 to 10 per 100,000 worldwide, commonly affecting individuals with risk factors such as congenital heart defects, prosthetic valves, intravenous drug use (IVDU), IV catheters, or recent dental infections [1,2-4]. IE can be classified based on the site of infection: left-sided native valve IE, right-sided native valve IE, left-sided prosthetic valve IE, and device-related (pacemaker or defibrillator) IE. An important aspect of the patient’s history is the mode of acquisition, whether community-acquired IE, nosocomial IE, or IVDU-associated IE [1]. The most common pathogens implicated in IE are *Staphylococcus aureus*, *Streptococcus* spp. (viridians, bovis), coagulase-negative *Staphylococcus*, and *Enterococcus* spp. [2,3]. Cases of gram-negative IE are scarce, and most of these organisms are non-*Pseudomonas*. IE associated with *Pseudomonas aeruginosa* (*P. aeruginosa*) is extremely rare, with many sources citing rates of 1.8% and 1.5%, with most cases involving IVDU [5]. We present a case of a male patient with a history of IVDU who presented with a relapse of *P. aeruginosa* bacteremia and native tricuspid valve (TV) endocarditis complicated by septic pulmonary emboli, despite undergoing recent vegetation debulking using the AngioVac system (AngioDynamics, Inc., New York, USA) along with six weeks of IV antibiotics and no IVDU since then.

## Case presentation

A 44-year-old male with a past medical history of HIV, hepatitis C virus, IVDU, and a recent hospitalization for TV endocarditis and bacteremia two months prior presented to our emergency department with the acute onset of sharp right upper abdominal pain, worse with inspiration, and associated with shortness of breath.

**Figure 1:**
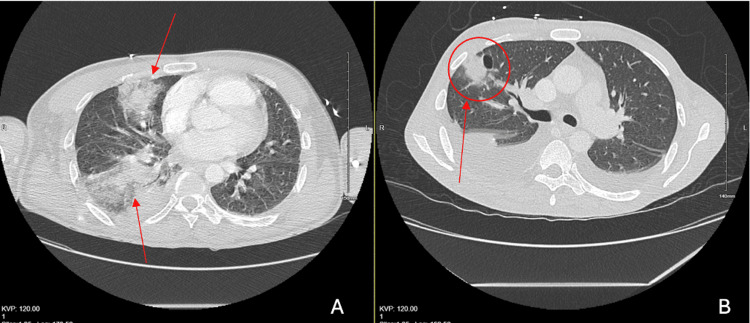
(A) A CT scan of the chest with contrast revealed acute right middle and lower lobar pulmonary emboli with nodular opacities (two red arrows) in the peripheral right upper lobe in the setting of recurrent TV endocarditis. (B) Compared with the CT from two months later, which revealed right middle lobe peripheral opacities (red arrow with circle) suspicious for septic emboli in the setting of TV endocarditis CT: computed tomography, TV: tricuspid valve

**Figure 2:**
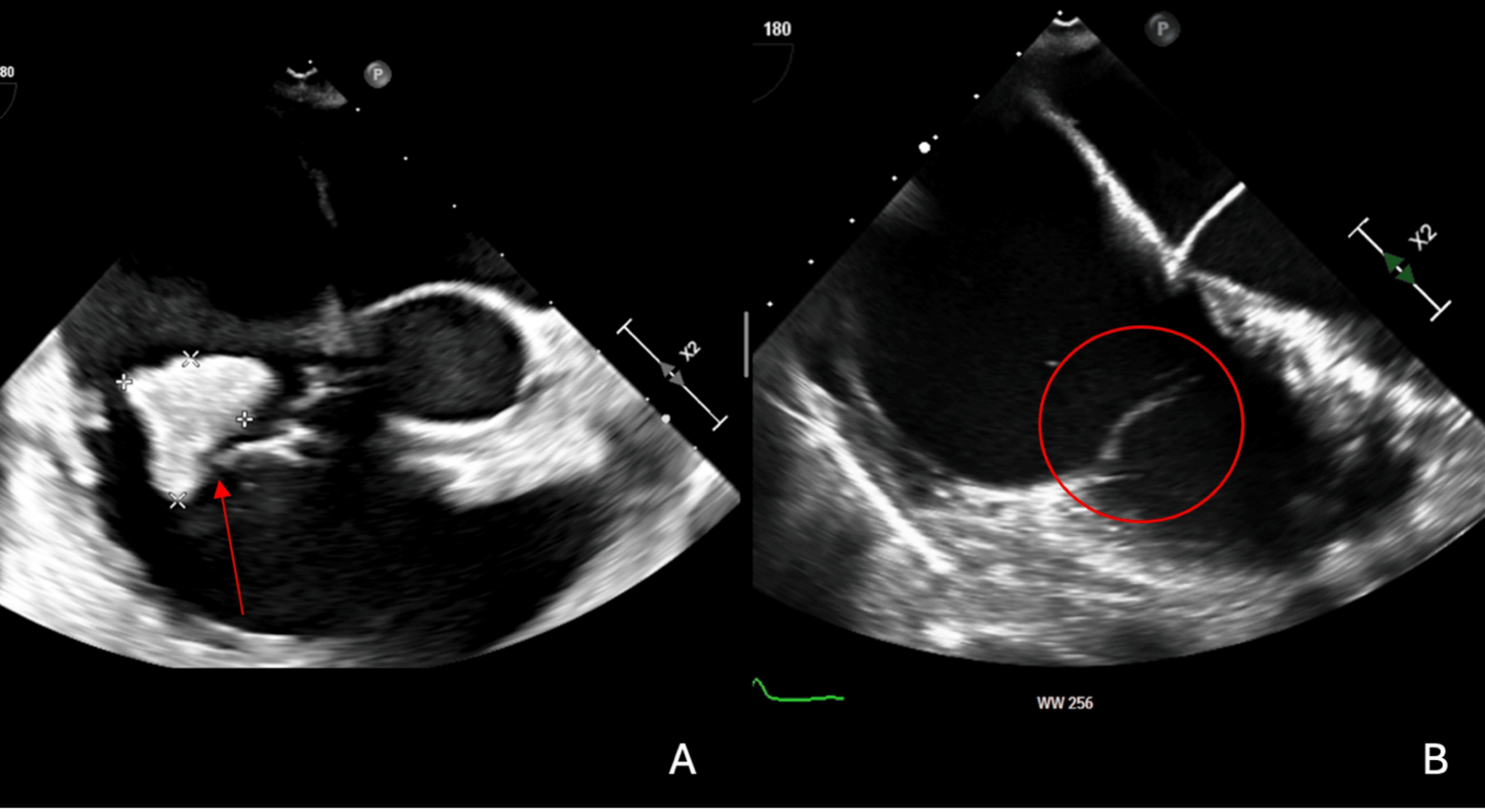
(A) TTE of the 2.09 cm x 1.8 cm large mobile vegetation between the anterior and posterior leaflets of the TV (red arrow). (B) TTE of the 0.63 cm x 0.49 cm on the tip of the posterior tricuspid leaflet on the atrium side (red circle). ‘A’ TTE from the initial visit; ‘B’ TTE from the second visit two months later TTE: transthoracic echocardiogram, TV: tricuspid valve

The patient originally presented to another hospital two months prior for a painful sacral ulcer and was not feeling well. The patient complained of a painful sacral decubitus ulcer for two weeks, unclear as to what caused it. The patient acknowledged a 20-pack-year cigarette smoking history and 30 years of drug use involving marijuana smoking, IV heroin use, intranasal cocaine, and smoking crack cocaine. Pt was found to have bacteremia with gram-negative rods and TV endocarditis and was transferred to our hospital for AngioVac. The patient was tachycardic at 103-125 bpm, tachypneic at 24-34 bpm, spiking low-grade fever of 100.4 F, and leukocytosis of 17,200/uL (Table 1). A CT scan of the chest with contrast revealed multiple peripherally located nodular opacities, some of which are cavitating and suspicious for septic emboli (Figure 1B). On a transthoracic echocardiogram (TTE), the patient had a 2.09 cm x 1.8 cm large mobile vegetation between the anterior and posterior leaflets of the TV and torrential tricuspid regurgitations secondary to leaflet perforation (Figure 2). The patient was initially started empirically on vancomycin (1 g, one dose), metronidazole (500 mg, every eight hours for one day), and cefepime (2 g, every 12 hours). Two of the two blood cultures grew *P. aeruginosa*, which was pan-sensitive to cefepime, levofloxacin, piperacillin-tazobactam, and tobramycin. Antibiotics were de-escalated to cefepime of only 2 g every eight hours. The patient underwent vegetation debulking using the AngioVac system without complications. A post-operative TTE indicated a small residual 0.9 cm x 0.9 cm mobile vegetation arising from the posterior leaflet. Heart tissue culture grew *P. aeruginosa*. Repeat blood cultures were negative. The patient was continued on cefepime 2 g every eight hours for a total of six weeks after a negative blood culture and discharged to subacute rehab with a peripherally inserted central catheter (PICC).

**Table 1:** Relevant laboratory findings of the initial hospitalization and the second hospitalization ESR: erythrocyte sedimentation rate, CRP: C-reactive protein

Laboratory finding	Value	Unit	Normal reference range
Heart rate (initial)	103-125	bpm	60-100 bpm
Respiratory rate (initial)	24-34	breaths/min	12-20 breaths/min
Temperature (initial)	100.4	°F	97.8-99.1 °F
Leukocyte count (initial)	17,200	/uL	4,000-11,000 /uL
Heart rate (second visit)	114-119	bpm	60-100 bpm
Respiratory rate (second visit)	27-39	breaths/min	12-20 breaths/min
Temperature (second visit)	98-99.7	°F	97.8-99.1 °F
Oxygen saturation (second visit)	92%	on room air	95-100%
Leukocyte count (second visit)	16,300	/uL	4,000-11,000 /uL
Neutrophil count	84%	%	40-60%
ESR	85	mm/hr	0-20 mm/hr
CRP	21.6	mg/dL	<0.29 mg/dL

Two weeks after completing the cefepime course, the patient presented to the emergency department, complaining of sharp right upper abdominal pain rated 8/10, worsened with inspiration, shortness of breath, and subjective fever for the past day. The patient denied using any IV drugs since the last hospital admission. The patient was normotensive, tachycardic at 114-119 bpm, tachypneic at 27-39 bpm, afebrile at 98-99.7 F, and hypoxic at 92% oxygen on room air (Table 1). The physical exam was remarkable for tenderness in the epigastric and right upper quadrants. Initial labs were significant for leukocytosis of 16,300/uL, with an 84% neutrophil count, an erythrocyte sedimentation rate of 85 mm/hr, and C-reactive protein of 21.6 mg/dL (normal range: <0.29 mg/dl in our institute) (Table 1). A bedside echocardiogram indicated right heart strain, but no vegetations were visible. A CT scan of the chest with IV contrast was remarkable for acute right middle and lower lobar pulmonary emboli of either septic or thrombotic origin and residual scarring and nodular opacities in the left lung base, along with other new superimposed ground-glass opacities and solid nodules that could suggest acute or chronic septic emboli (Figure 1A). An ultrasound of the bilateral lower extremities indicated no evidence of deep vein thrombosis. TTE revealed severe TV regurgitation, and a follow-up transesophageal echocardiogram (TEE) revealed a 0.63 cm x 0.49 cm vegetation on the tip of the posterior tricuspid leaflet on the atrium side. Two of the two blood cultures grew *P. aeruginosa*, which was pan-sensitive to cefepime, levofloxacin, piperacillin, tazobactam, and tobramycin. The patient was treated initially with dual coverage using meropenem (2 g every eight hours) and tobramycin and later de-escalated to meropenem only. Repeat blood cultures a few days later were negative for any growth, and leukocytosis normalized. The cardiothoracic surgery team completed the TV replacement without complications. Culture from the heart tissue expectedly grew pan-sensitive *P. aeruginosa*. Repeat blood cultures did not grow any organisms. The patient received a PICC line and was discharged to a subacute rehab facility for six weeks of IV meropenem from the date of the valve replacement procedure.

## Discussion

Here, we present an interesting case of a patient who developed TV endocarditis caused by *P. aeruginosa* bacteremia complicated by septic emboli to the lungs and failed six weeks of appropriate antimicrobial therapy and AngioVac, leading to tricuspid valve replacement.

The most common organisms implicated in IE are *Staphylococcus aureus*, *Streptococcus* spp., and *Enterococcus*. Others include *Haemophilus* spp.*, Aggregatibacter actinomycetemcomitans*, *Cardiobacterium hominis*, *Eikenella corrodens*, and *Kingella kingae* (HACEK group). IE caused by non-HACEK gram-negative bacteria, such as *P. aeruginosa*, is extremely rare, with some sources citing less than 1.8% and 1.5% [5-6,7]. *P. aeruginosa* IE is typically seen in IVDU since it is commonly associated with contaminated water, which drug users may use to mix certain drugs to clean their wounds and needles. In general, left-sided IE is associated with increased mortality due to the danger of embolism to the brain, compared to right-sided IE, which may cause pulmonary embolism.

The latest recommendation for treating non-HACEK IE includes early surgery and at least six weeks of antibiotic treatment with a combination of β-lactams and aminoglycosides [1]. Mortality is significant (24%) and requires optimal management on a case-by-case basis with an infectious disease specialist since there is limited trial data on the most optimal antimicrobial combination for non-HACEK gram-negative endocarditis [1,4]. Fifty to seventy-five percent of *P. aeruginosa* right-sided cases are successfully managed medically without the need for surgical interventions [4]. However, patients who fail the initial course of medical therapy should undergo vegetation removal using less invasive means before any valve replacement is considered [4,5]. Placing a prosthetic valve in an IVDU patient should be the last resort since there is a high relapse rate and a prosthetic valve acts as a nidus for future vegetations, increasing the risk for recurrent endocarditis episodes [4,5,8-9]. The recommendations for early valve surgery depend on whether it is left-sided or right-sided, on a prosthetic valve or native valve, and on a prior history of emboli or stroke. For right-sided IE, there is an association with IVDU; thus, it is recommended to not place prosthetic valves and manage patients medically [4,10-11]. However, surgical intervention is reasonable if the following indications are present: right heart failure secondary to severe tricuspid regurgitation, tricuspid vegetations over 20 mm, recurrent pulmonary embolism despite treatment, and sustained infection (especially with multidrug-resistant organisms) despite treatment [4,10-11].

During the initial hospitalization, our patient was empirically treated with vancomycin, metronidazole, and cefepime until blood culture results returned. Two out of two blood cultures grew only *P. aeruginosa,* and TEE revealed a large 2.09 cm x 1.8 cm mobile vegetation between the anterior and posterior leaflets of the TV, with septic emboli to the lungs on a CT scan. The cardiothoracic team weighed all management options and decided on vegetation debulking using the AngioVac system along with medical therapy (cefepime). Since the vegetation was larger than 20 mm, resulting in severe tricuspid regurgitation and septic pulmonary emboli, it was determined that the most ideal management is the AngioVac system rather than tricuspid valve replacement as the patient had a long history of IVDU and would predispose him to future episodes of endocarditis. However, there was a small residual 0.9 cm x 0.9 cm mobile vegetation at the posterior leaflet.

The AngioVac system was approved in 2014 for the removal of materials within the intravascular system through percutaneous means [12]. Although numerous reports exist with regard to the use of the AngioVac device in aspiration of iliocaval, pulmonary, upper extremity, and right-sided heart chamber thrombi, very few data are present demonstrating its use in the treatment of right-sided endocarditis. Data is limited when it comes to its use in right-sided cardiovascular thrombi; however, it is a great option for patients who are poor surgical candidates and IVDU who would benefit from it before any valve replacements [12]. Our patient tolerated the AngioVac procedure for large TV anterior leaflet vegetation measuring 2.09 cm x 1.8 cm. Data on the use of AngioVac for right-sided IE management is limited, but in a 2017 retrospective study of 33 patients with TV IE with an average vegetation size of 2.1 ± 0.7 cm and a post-procedure size of 0.82 ± 0.5 cm, there was a 90.9% survival rate and no reinfection after the AngioVac procedure [13]. The 2017 retrospective study of 33 patients has its limitations as the sample size is small, and 25 of those patients had *Staphylococcus aureus* and five had fungemia. On average, there was a 61% reduction in vegetation size after the AngioVac with 0.82 ± 0.5 cm remaining, similar to our patient with 0.9 cm x 0.9 cm vegetation after the procedure [13]. Vegetation size was deemed a major prognostic factor, as vegetations over 2 cm were associated with a mortality rate of 25% [13]. Although small vegetation may be left behind after AngioVac, it still allows for a major reduction in the bacterial load burden, hence increasing the efficacy of the antibiotics [12,13].

A compilation of literature database searches, via PubMed and Embase, on the use of AngioVac in right-sided IE was tabulated and summarized in a 2017 article [12]. Most of the literature included case reports and case series, with a 2017 retrospective study of 33 patients and a 2016 retrospective study of 20 patients with implantable cardioverter defibrillators or pacemakers [12,13]. Most of the patients in all of these cases reported had methicillin-sensitive *Staphylococcus aureus*, methicillin-resistant *Staphylococcus aureus*, and a few *Candida*, *Enterococcus*, and polymicrobial but lacking cases of *P. aeruginosa* [13]. A 2022 comprehensive systematic review and meta-analysis, including 44 studies with 301 patients, was conducted to evaluate the efficacy and safety of AngioVac vegetation debulking in right-sided IE [14]. Procedural success (residual vegetation size <50% without serious angiographic complications) was achieved in 89.2% of patients (95% CI: 82.3-93.6%, I2=0%).

Clinical success (residual vegetations <50%, in-hospital survival, absence of recurrent bacteremia, and valve functions not requiring further interventions) was achieved in 79.1% of patients (95% CI: 67.9-87.2%, I2=15%). Overall survival rate was 89.7% (95% CI: 83.1-93.9%%, I2=9%), and clearance of bacteremia was 82.5%. This meta-analysis demonstrates that AngioVac is a promising therapeutic option for RSIE, offering a high success rate with an acceptable complication rate across a wide range of patients. However, data regarding *P. aeruginosa* tricuspid endocarditis remains extremely limited. It is possible that in our patient, the residual vegetation measuring 0.9 cm on the posterior leaflet was enough to cause a pulmonary septic embolus even after six weeks of cefepime and acted as a nidus for the patient’s relapse endocarditis less than two months later. During the second hospitalization, the patient was found to be septic, hypoxic, with leukocytosis, and had vegetation on the TV measuring 0.63 cm x 0.49 cm on the tip of the posterior. Could the patient have benefited from a TV repair or replacement during the initial hospitalization, especially since the AngioVac did not remove all the vegetations? However, most of the limited literature recommends less invasive methods before any surgical management in IVDU. Once our patient was stable, the cardiothoracic team opted to proceed with a TV replacement, even though the patient is an IVDU and will remain at an increased risk for future endocarditis. It was deemed the best decision after failing the initial treatment.

A contemporary, retrospective case series review identified a total of 15 cases over a 20-year period (1999-2019) with *P. aeruginosa* and IE, meeting the modified Dukes criteria [15]. A prior history of IE was in 2/15 (13%), IVDU in 6/15 (40%), TV involvement in 3/15 (20%), and major emboli in 3/15 (20%) [15]. Treatment choices varied depending on isolate susceptibility, ranging from meropenem, cefepime, piperacillin-tazobactam, and ciprofloxacin, but at least six weeks of IV antibiotics were administered in all cases surviving over 30 days [15]. Only two cases out of the 15 had a relapse after treatment, also with *P. aeruginosa*, and both cases were also IVDU and involved native valves [15]. In one of the relapse cases, the patient had TV involvement and was initially treated with six weeks of meropenem and gentamicin without any surgical intervention, but relapsed, so he was treated for six more weeks of meropenem monotherapy and was alive a year later [15]. The second case of relapse was a patient with mitral valve involvement who was treated with piperacillin-tazobactam; however, the patient developed relapse due to piperacillin-tazobactam resistance and was treated with mitral valve surgical repair and six weeks of meropenem; the patient was alive five years later [15]. Our case is unique because the patient was treated with AngioVac and six weeks of IV cefepime but relapsed, and the isolate remained pan-sensitive to all antibiotics, including cefepime. During the relapse hospitalization, the patient received a TV replacement and was discharged to a rehab facility for six weeks of IV meropenem. The patient was instructed to continue follow-up with infectious disease and cardiothoracic surgery to monitor progress.

## Conclusions

*P. aeruginosa* continues to be a rare cause of IE and is on the rise as IVDU rates increase. We highlight the unique case of bacteremia with *P. aeruginosa* and TV endocarditis, initially treated with AngioVac and cefepime for six weeks, cleared bacteremia, readmitted with bacteremia relapse with *P. aeruginosa* that remained pan-sensitive to all antibiotics, and complicated by pulmonary septic emboli and persistent TV endocarditis with severe TV regurgitation. There is very limited literature and cases of *P. aeruginosa* IE, or the efficacy of AngioVac on right-sided vegetation debulking, and there is a need for future studies to weigh the benefits and risks of valve replacement compared to the less invasive AngioVac system in IVDU.
